# Expression of ALDH1 in breast invasive ductal carcinoma: an independent predictor of early tumor relapse

**DOI:** 10.1186/1475-2867-13-60

**Published:** 2013-06-15

**Authors:** Ying Zhong, Yan Lin, Songjie Shen, Yidong Zhou, Feng Mao, Jinghong Guan, Qiang Sun

**Affiliations:** 1 Department of Breast Disease, Peking Union Medical College Hospital, Peking Union Medical College, Beijing, 100730, China

**Keywords:** Aldehyde dehydrogenase 1, Prognosis, Breast, Invasive ductal carcinoma

## Abstract

**Background:**

The specific mechanism underlying the contribution of the Aldehyde dehydrogenase 1 (ALDH1) phenotype to metastatic behavior and early tumor relapse in breast cancer is currently unclear.

**Methods:**

147 randomly selected invasive ductal carcinoma samples were assayed for expression of ALDH1A1, NOTCH1, estrogen receptor (ER), progesterone receptor (PR), and human epidermal growth factor receptor (HER2), and association of the ALDH1A1 phenotype with clinic pathological features was further evaluated.

**Results:**

ALDH1A1-positive cells were detected in 63.3% (93 of 147) of tumors. 80.0% (32 of 40) of tumors with strong ALDH1A1 staining displayed early recurrence, compared with 20.0% (8 of 40) of tumors negative for ALDH1A1 expression (*P* = 0.027). ALDH1A1 status was significantly correlated with strong malignant proliferative marker Ki67 staining (*P* = 0.001), and no significantly different expression of ALDH1A1 across the subtypes of ER, PR, and HER2 expression and triple negative features of tumor tissue. Multivariate regression analysis demonstrated that elevated ALDH1A1 expression is an independent predictor of recurrence-free survival and distant metastasis-free survival. Notably, breast cancer tissue strong for ALDH1A1 expression displayed weak NOTCH1 staining compared to ALDH1A1 weak tumor tissue (*P* = 0.002), and the relationship between ALDH1A1 and NOTCH1 mRNA positivity was significant (Pearson correlation - 0.337, *P* = 0.014; Spearman’s rho - 0.376, *P* = 0.006). Elevated NOTCH1 mRNA level (using a cut-off value based on the median ALDH1A1 2^-△△*C*T^ value) was associated with reduction of ALDH1A1 mRNA level (*P* = 0.001).

**Conclusions:**

The ALDH1A1 phenotype is an independent predictor of early tumor relapse characteristic (specifically, incidence of early local recurrence and distant metastasis) of invasive ductal carcinoma. The NOTCH1 signaling pathway is possibly involved in the negative association of the ALDH1A1 phenotype with early malignant relapse in invasive ductal carcinoma.

## Introduction

Breast invasive ductal carcinoma is a common breast malignancy and a major cause of cancer-related death in women worldwide [1]. Despite developments in surgical methods, cytotoxic chemotherapy, and targeting agents against estrogen receptor and HER2, a subset of patients with advanced-stage invasive ductal carcinoma display poor prognosis and early metastasis after single or combination treatment. An estimated 11% of women with invasive ductal carcinoma will experience recurrence within five years after surgery, including 8% with luminal A breast cancers and 15% with triple negative tumors [2,3].

The cancer stem cell hypothesis was proposed to explain breast cancer heterogeneity and risk of recurrence. These cell subpopulations have the capacity to self-renew and differentiate into multiple cell types, and may contribute to drug resistance that promotes tumor recurrence or metastasis [4]. Several cellular subcomponent changes have been described in breast cancer, including aldehyde dehydrogenase 1 (ALDH1) positivity, CD44 positivity, CD24 negativity, RHOC overexpression, hypomethylation of caveolin promoters, and deletion of some tumor suppressors [5-9]. Among these molecules, ALDH1, an enzyme responsible for the oxidation of intracellular aldehydes, has been a subject of research focus in recent years [9,10]. Several studies have suggested that ALDH1 contributes to normal and tumor stem cell differentiation, and invasion and metastasis in breast cancer are mediated by a cellular subcomponent with stem cell characteristics expressing ALDH1 [11,12]. For example, populations of normal mammary epithelial cells with increased ALDH1 activity have the ability to form mammospheres and self-renew, and breast carcinoma cells with high ALDH1 activity display tumor-generating potential. These findings indicate that the breast carcinoma cells with ALDH1 phenotype participate in the acquisition of progenitor features [9,13]. In addition, emerging evidence suggests that ALDH1 plays important functional roles related to self-protection [14]. Another previous report describing the association of ALDH1 expression with early metastasis and decreased survival in inflammatory breast cancer has further demonstrated a critical role of ALDH1-positive cancer cells in mediating the clinically aggressive behavior of breast cancer [15]. However, the mechanisms by which the ALDH1 phenotype contributes to malignant cell metastatic behavior, such as early tumor relapse, distant recurrence, self-renewal, and proliferation in breast cancer are yet to be established.

In terms of regulation of cellular proliferation and differentiation, several known signaling pathways, such as NOTCH, play a role in self-renewal of stem cells [16,17]. Previously, up regulation of NOTCH ligands led to elevation of the mammosphere number, and conversely, down regulation abrogated mammosphere formation, providing evidence that the NOTCH signal pathway contributes to mammary gland development [18]. On the other hand, although overexpression of NOTCH ligands in a transgenic mouse model triggered breast cancer, supporting the theory that NOTCH contributes to cancer development [19,20], the finding that NOTCH signaling is diminished in some solid tumors would seem to suggest that NOTCH might serve as a tumor suppressor [21,22]. However, no evidence of an association of the NOTCH signaling pathway with proliferation or suppression of the ALDH1-expressing cellular subcomponent displaying early tumor relapse characteristics has been obtained to date.

In the current study, we primarily investigated whether breast cancer cells with the ALDH1 phenotype contribute to early malignant relapse behavior, and further discussed the possible underlying biological mechanisms.

## Materials and methods

### Patients and specimens

In total, 147 invasive ductal carcinoma samples were randomly selected from our tissue database of patients treated at the Peking Union Medical College Hospital between April 2000 and December 2007. None of the patients had received neoadjuvant chemotherapy or radiotherapy. Clinical information was obtained by reviewing preoperative and perioperative medical records, follow-up records, and written correspondence. Patients were staged based on tumor-node-metastasis (TNM) classification of the International Union Against Cancer, revised in 2002 [23]. The clinical characteristics of patients are shown in Table 1. Fresh-frozen tumor tissue samples were used for routine examination of the estrogen receptor (ER), progesterone receptor (PR), and human epidermal growth factor receptor (HER) 2. Paraffin specimens of these tumors were collected, and 5 mm-thick tissue sections cut and fixed onto silicified slides. Each sample was stained with hematoxylin and eosin (H&E), and histologically typed according to the World Health Organization (WHO) classification system [24]. Tumor sizes, and the number and location of metastatic lymph nodes were obtained from pathology reports. The use of human materials was approved by the Peking Union Medical College Hospital Medical Ethics Committee (Full name of the board/committee: Peking Union Medical College Hospital Medical Ethics Committee. No.S-294). We confirm that written informed consent from the donor or the next of kin was obtained for use of this sample in research.

**Table 1:** **Association of ALDH1 expression with clinical and pathologic factors in breast cancer tissues (*χ***^**2 **^**test)**

	**n**	**ALDH1A1 expression**				***P *value**
		**–**	**+**	**++**	**+++**	
Age (years)	147	52.2 ± 11.9	51.6 ± 13.0	53.2 ± 13.0	48.5 ± 12.7	0.682
Tumor size (cm)	147	3.9 ± 0.6	2.6 ± 0.2	2.9 ± 0.6	3.9 ± 0.5	0.103
Lymph node involvement	115	45 (39.1%)	45 (39.1%)	9 (4.0%)	16 (13.9%)	0.389
TNM stages						
I	14	4 (28.6%)	8 (57.1%)	1 (7.1%)	1 (7.1%)	0.177
II	56	19 (33.9%)	25 (44.6%)	6 (10.7%)	6 (10.7%)	
III	76	31 (40.8%)	29 (38.2%)	5 (6.6%)	11 (14.5%)	
IV	1	0 (0.0%)	0 (0.0%)	0 (0.0%)	1 (100.0%)	
NOTCH1 expression						
–	9	7 (77.8%)	2 (22.2%)	0 (0.0%)	0 (0.0%)	0.044
+	26	7 (26.9%)	17 (65.4%)	2 (7.7%)	0 (0.0%)	
++	24	10 (41.7%)	8 (33.3%)	1 (4.2%)	5 (20.8%)	
+++	88	30 (34.1%)	36 (40.9%)	8 (9.1%)	14 (15.9%)	
Ki67 expression						
–	79	38 (48.1%)	35 (44.3%)	6 (7.6%)	0 (0.0%)	0.001
+	68	16 (23.5%)	28 (41.2%)	5 (7.4%)	19 (27.9%)	
P53 expression						
–	100	35 (35.0%)	45 (45.0%)	8 (8.0%)	12 (12.0%)	0.823
+	47	19 (40.4%)	18 (38.3%)	3 (6.4%)	7 (14.9%)	
ER expression						
–	90	34 (37.8%)	35 (38.9%)	6 (6.7%)	15 (16.7%)	0.303
+	57	20 (35.1%)	28 (47.4%)	5 (8.8%)	4 (7.0%)	
PR expression						
–	83	27 (32.5%)	38 (45.8%)	5 (6.0%)	13 (15.7%)	0.400
+	64	27 (42.2%)	25 (39.1%)	6 (9.4%)	6 (9.4%)	
HER2 expression						
–	77	32 (41.6%)	32 (41.6%)	4 (5.2%)	9 (11.7%)	0.492
+	70	22 (31.4%)	31 (44.3%)	7 (10.0%)	10 (14.3%)	
Triple negativity features^*^						
–	108	40 (37.0%)	44 (40.7%)	10 (9.3%)	14 (13.0%)	0.541
+	39	14 (35.9%)	19 (48.7%)	1 (2.6%)	5 (12.8%)	
Local recurrence						
Present	40	8 (20.0%)	20 (50.0%)	6 (15.0%)	6 (15.0%)	0.027
Absent	107	46 (43.0%)	43(40.2%)	5 (4.7%)	13 (12.1%)	
Distant metastasis						
Present	51	21 (41.1%)	20 (39.2%)	3 (5.9%)	7 (13.7%)	0.809
Absent	96	33 (34.3%)	43 (44.8%)	8 (8.3%)	12 (12.5%)	

*ALDH* aldehyde dehydrogenase, *ER* estrogen receptor, *PR* progesterone receptor, *HER2* human epidermal growth factor receptor 2.

^*^ Immunohistochemically negative for ER, PR, and HER2.

### Immunohistochemical staining and evaluation

Briefly, individual tissue sections were deparaffinized, rehydrated and incubated with fresh 3% hydrogen peroxide (H_2_O_2_) in methanol for 15 min. After rinsing with phosphate-buffered saline (PBS), samples were immersed in 0.01 M sodium citrate buffer (pH 6.0) and heated in a microwave oven at 100 °C for 15 min for antigen retrieval. Non-specific binding was blocked by incubating the sections with normal goat serum for 15 min at room temperature. Samples were subsequently incubated at 4°C overnight with different primary antibodies, including rabbit monoclonal to ALDH1 (ALDH1A1, IgG, 1:100, Abcam, Cambridge, UK), rabbit polyclonal to NOTCH1 (NOTCH1, IgG, 1:100, Abcam, Cambridge, UK), FITC-linked mouse monoclonal to SABC (1:50), and goat anti-rabbit Cy3 antibody (IgG, 1:20). ALDH1 and NOTCH1 expression were detected using a Nikon Eclipse 80i microscope and the Mcv2000 Image Analysis System. All slides were counterstained with hematoxylin to identify nuclei. Samples were scored twice by one individual in a blinded fashion, and unclear findings were further discussed with a pathologist. In cases of staining discrepancies among the three cores from the same patient, an average value was used. ALDH1 and NOTCH1 staining were detected mainly in the cytoplasm.

### Real-time PCR

Total RNA was obtained using the RNAqueous-Micro kit (Ambion, Austin, TX, USA), following the manufacturer’s instructions. Power SYBR Green PCR Master Mix (Applied Biosystems) was employed to amplify the corresponding genes with primers specific for human NOTCH 1 (forward: 5'-GACCTCATCAACTCACACGC-3', reverse: 5'-CGGCATCCACATTGTTCA-3'). Human GAPDH (Hs_GAPDH_2_SG QuantiTect Primer Assay QT01192646, Qiagen, Hilden, Germany) was used as the loading control. Real-time PCR was performed on a Linegene Real-time PCR detection system (Bioer Technology, China). Data were analyzed using the 2^-△△*C*T^ method [25].

### Statistical analysis

All calculations were performed using SPSS V.14.0 statistical software (Chicago, IL, USA). *T*-test, Mann–Whitney *U* test, and Fisher’s exact test were applied where appropriate. The Kaplan-Meier method was used to estimate cumulative recurrence-free survival (RFS) and metastasis-free survival (MFS), and the log-rank test to compare survival between two strata, respectively. All tests were two-sided, and *P* < 0.05 was considered statistically significant.

## Results

### Patient characteristics

The baseline characteristics of the study population are presented in Table 1. All patients were female, with a mean age of 51.6 ± 12.5 years (range, 13.5 to 80.7 years) and mean tumor size of 3.1 ± 1.8 cm (range, 0.4 to 9.5 cm). Lymph node involvement was positive in 115 patients (78.2%). According to the TNM staging system, 14 patients (9.5%) were classified as stage I, 56 (38.1%) as stage II, 76 (51.7%) as stage III, and 1 (0.7%) as stage IV. Among the 147 patients, 57 (38.8%) were positive for ER, 64 (43.5%) for PR, 70 (47.6%) for HER2, and 39 (26.5%) for triple negativity features (defined as immunohistochemically negative for both SR and HER2). Median follow-up time was 23.0 months (range, 2 to 91 months), during which 27.2% patients (40 of 147) experienced tumor recurrence and 34.7% (51 of 147) developed metastases.

### Presence of the ALDH1A1 phenotype in invasive ductal carcinoma tissue

**Figure 1:**
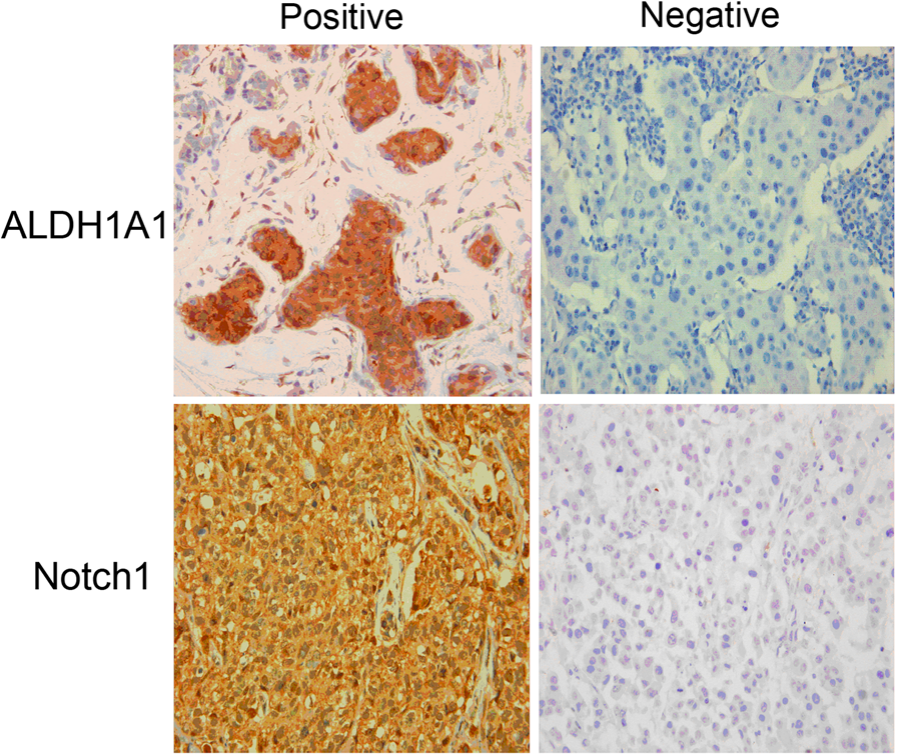
Immunohistochemical analysis of ALDH1A1 and NOTCH1 expression in invasive ductal carcinoma samples.

Immunohistological analysis of serial tumor sections revealed ALDH1A1 positivity in cells from invasive ductal carcinoma tissues, as illustrated by strong cytoplasmic staining (Figure 1). ALDH1A1-positive cells were detected in 63.3% (93 of 147) of tumors, with 42.9% (63 of 147) showing slight staining, 7.5% (11 of 147) moderate staining, and 12.9% (19 of 147) strong staining. Furthermore, in the follow-up period, 80.0% (32 of 40) of tumors with positive ALDH1A1 expression displayed recurrence, compared with 20.0% (8 of 40) of ALDH1A1-negative tumors (*P* = 0.027). ALDHA1-negative cells were mainly observed in the cases without local recurrence (43.0%, 46/107 cases). On the other hand, no linkage was observed between ALDH1A1 phenotype and postoperative metastasis. Moreover, we observed no stepwise increase in the prevalence of ALDH1A1 expression with TNM stage, lymph node involvement, ER, PR, and HER2 expression and triple negativity features of invasive ductal carcinoma tissue, it demonstrated that no significantly different expression of ALDH1 across these subtypes in invasive ductal carcinoma (Table 1).

### Association of the ALDH1A1 phenotype with RFS and MFS

**Figure 2:**
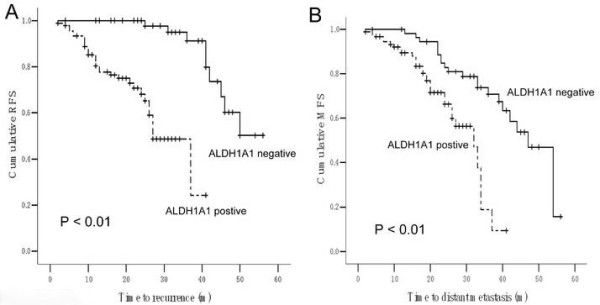
Analysis of recurrence-free survival (RFS, A) and distant metastasis-free survival (MFS, B) in breast cancer patients with and without the ALDH1 phenotype.

Local recurrence-free survival differed significantly between ALDH1A1 subtypes. Median RFS in ALDH1A1 positive tumors was 28.1 months (95% CI: 24.8–31.4) compared with 49.3 months (95% CI: 45.8–52.7) in ALDH1A1 negative tumors (*P* = 0.001, Figure 2A). Meanwhile, distant metastasis-free survival also showed prominent difference between ALDH1A1 subtypes. Median MFS in ALDH1A1 positive tumorscpe was 27.7 months (95% CI: 25.0–30.5) compared with 43.2 months (95% CI: 39.2–47.2) in ALDH1A1 negative tumors (*P* = 0.001, Figure 2B). Our data demonstrated that positive ALDH1A1 phenotype was significantly associated with RFS and MFS. Notably, multivariate Cox proportional hazards regression analysis implied that elevated ALDH1A1 expression in invasive ductal carcinoma is an independent predictor of recurrence-free survival and also distant metastasis-free survival (Tables 2 and 3). Therefore, the ALDH1A1 phenotype is an independent predictor of early tumor relapse characteristic (specifically, incidence of local recurrence and distant metastasis) of invasive ductal carcinoma.

**Table 2:** Univariate and multivariate analysis of ALDH1A1 phenotype in relation to recurrence-free survival (RFS)

**Variable**	**Univariate analysis**			**Multivariate analysis**		
	**HR**	**95% CI**	***P *value**	**HR**	**95% CI**	***P *value**
ALDH1A1						
Positive	11.932	4.190–33.977	0.001	11.399	3.776–34.414	0.001
Negative	1.000			1.000		
ER status						
Positive	1.013	0.519–1.977	0.970	1.579	0.636–3.921	0.325
Negative	1.000			1.000		
PR status						
Positive	0.669	0.345–1.299	0.235	0.568	0.238–1.358	0.203
Negative	1.000			1.000		
HER2 status						
Positive	1.307	0.701–2.435	0.400	1.564	0.599–4.085	0.361
Negative	1.000			1.000		
Triple negativity features*						
Present	0.848	0.441–1.631	0.620	0.808	0.241–2.702	0.729
Absent	1.000			1.000		
TNM stage						
Stage III/IV	0.718	0.373–1.382	0.321	0.803	0.402–1.601	0.532
Stage I/II	1.000			1.000		
Age (years)						
≥ 50	0.709	0.376–1.338	0.289	0.803	0.402–1.601	0.532
< 50	1.000			1.000		

Abbreviations: *HR* hazard ratio estimated from Cox proportional hazard regression model, *CI* confidence interval of the estimated HR, *ER* estrogen receptor, *PR* progesterone receptor, *HER2* human epidermal growth factor receptor 2.

^*^Immunohistochemically negative for ER, PR, and HER2.

**Table 3:** Univariate and multivariate analysis of ALDH1A1 phenotype in relation to distant metastasis-free survival (MFS)

**Variable**	**Univariate analysis**			**Multivariate analysis**		
	**HR**	**95% CI**	***P *value**	**HR**	**95% CI**	***P *value**
ALDH1A1						
Positive	3.501	1.785–6.866	0.001	3.562	1.790–7.091	0.001
Negative	1.000			1.000		
ER status						
Positive	1.111	0.614–2.012	0.727	1.510	0.693–3.290	0.300
Negative	1.000			1.000		
PR status						
Positive	0.956	0.546–1.674	0.875	1.052	0.479–2.310	0.900
Negative	1.000			1.000		
HER2 status						
Positive	0.969	0.554–1.694	0.911	1.161	0. 540–2.495	0.703
Negative	1.000			1.000		
Triple negativity features*						
Present	0.870	0.481–1.573	0.645	0.741	0.265–2.073	0.569
Absent	1.000			1.000		
TNM stage						
Stage III / IV	1.874	1.010–3.475	0.046	2.248	1.173–4.307	0.015
Stage I / II	1.000			1.000		
Age (years)						
≥ 50	1.399	0.793–2.467	0.247	1.585	0.846–2.970	0.151
< 50	1.000			1.000		

Abbreviations: *HR* hazard ratio estimated from Cox proportional hazard regression model, *CI* Confidence interval of estimated HR. *ER* estrogen receptor, *PR* progesterone receptor; *HER2* human epidermal growth factor receptor 2.

^*^Immunohistochemically negative for ER, PR, and HER2.

### Association of the ALDH1A1 phenotype with proliferative features

Among the 147 samples with available data on Ki67, one of the malignant proliferative indices, 51.9% (41 of 79) cases with negative Ki67 expression and 76.5% (52 of 68) with positive Ki67 expression were positive for the ALDH1A1 phenotype, respectively. ALDH1 status was significantly correlated with strong Ki67 staining in all patients (*P* = 0.001), indicating an association of the ALDH1A1 phenotype with malignant proliferation in invasive ductal carcinoma.

### Association of the ALDH1A1 phenotype with NOTCH1 mRNA

**Figure 3:**
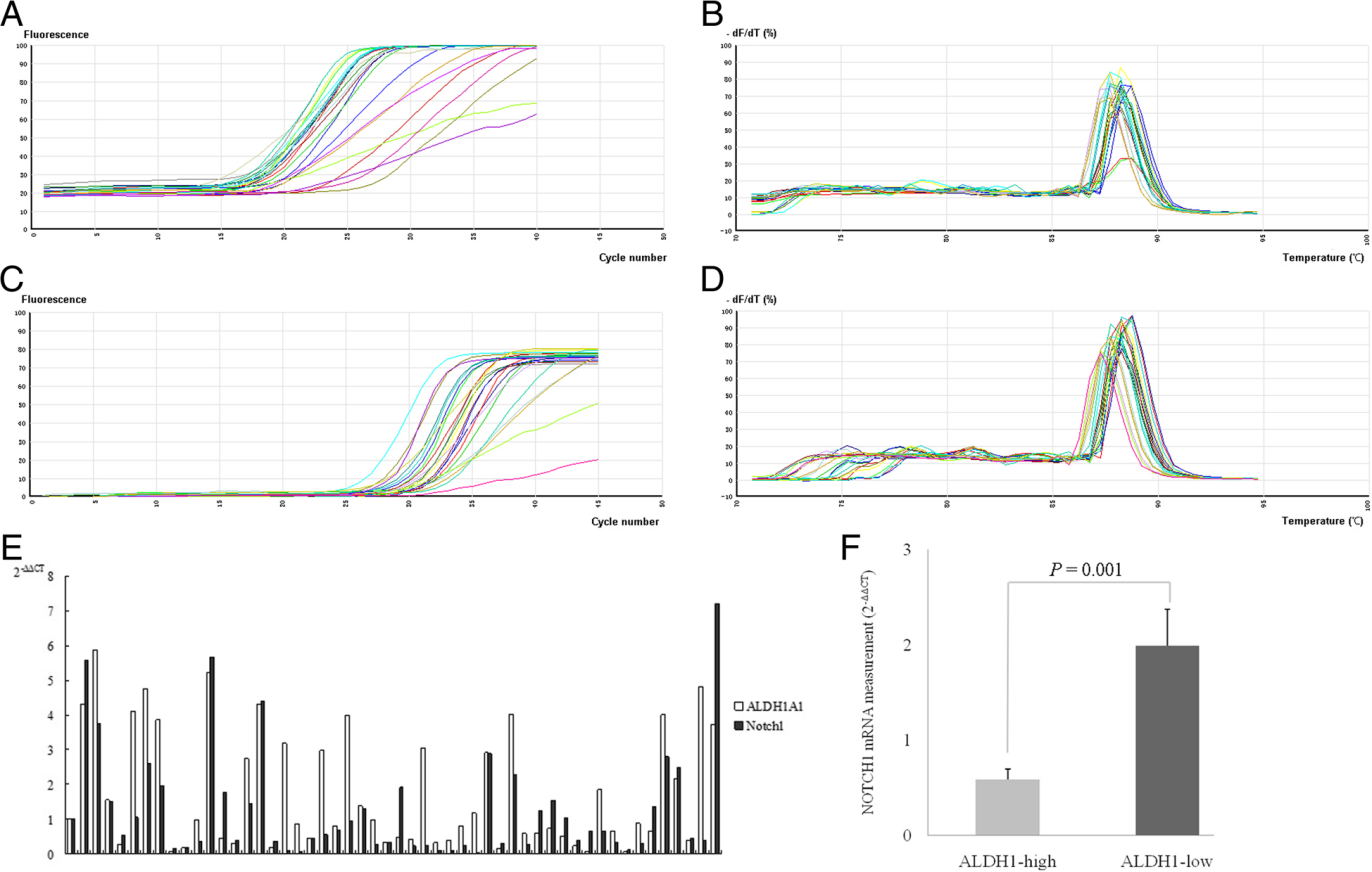
**Results of real-time PCR. A**. Amplified curve analysis of actin. **B**. Melting curve analysis of actin. **C**. Amplified curve analysis of NOTCH1. **D**. Melting curve analysis of NOTCH1. **E**. Compare of 2^-△△*C*T^ value of ALDH1A1 and NOTCH1 expression in each case. **F**. Compare of mean 2^-△△*C*T^ value of NOTCH1 expression regarding to ALDH1A1 expression (using a cut-off value based on the median ALDH1A1 2^-△△*C*T^ value).

In immunohistochemical experiments, we observed a stepwise decrease in the prevalence of ALDH1A1 expression with NOTCH1 status (*P* = 0.044) (Table 1, Figure 1). ALDH1A1-negative breast cancer tissue displayed strong NOTCH1 staining (1.92 ± 0.37), compared to ALDH1A1-positive breast cancer tissue (0.61 ± 0.11, *P* = 0.002). Real-time PCR experiments revealed a significant relationship between ALDH1A1 and NOTCH1 mRNA in 52 samples (Pearson correlation - 0.337, *P* = 0.014; Spearman’s rho - 0.376, *P* = 0.006, Table 4). It seemed many high ALDH1A1 mRNA samples showed weak NOTCH1 mRNA level, and moderate or high expression of NOTCH1 was parallel to the absence or little expression of ALDH1 expression (Figure 3E). Elevated NOTCH1 mRNA level (using a cut-off value based on the median ALDH1A1 2^-△△*C*T^ value) was associated with reduction of ALDH1A1 mRNA level (*P* = 0.001, Figure 3F). Our findings collectively suggest a possible negative association of the ALDH1A1 phenotype with NOTCH1 in invasive ductal carcinoma.

**Table 4:** **Measurement of ALDH1A1 and NOTCH1 mRNA with real-time PCR**^***a***^

**Target**	**ALDH1A1**	**NOTCH1**	**β-actin**
*n*	52	52	52
Avg. C_T_	22.55 ± 1.21 (19.25~25.07)	28.12 ± 1.65 (24.62~31.76)	18.19 ± 2.16 (14.63~22.80)
△C_T_	−1.03 ± 0.21 (− 4.33~1.49)	−0.50 ± 0.65 (− 4.01~3.14)	−1.11 ± 0.16 (− 4.67~3.50)
△△C_T_	0.08 ± 0.01 (− 2.56~4.37)	0.61 ± 0.03 (− 2.85~4.49)	
2^-△△CT^	1.75 ± 0.70 (0.05~5.88)	1.29 ± 0.58 (0.04~7.19)	

^*a*^ Total RNA was purchased from Clontech, and cDNA synthesized from 1 mg total RNA using reverse transcriptase. Aliquots of cDNA were used as the template for real-time PCR reactions containing either primers and probe for ALDH1A1 or NOTCH1 and probe for β-actin. Each reaction included cDNA derived from 10 ng total RNA, and six replicates per reaction were performed.

## Discussion

The important properties of cancer stem-like cells include *in vitro* self-renewal, *in vivo* tumor initiation, and generating a heterogeneous population of cancer cells. Proliferation is an important characteristic in tumor biology. ALDH1-positive cancer cells are highly clonogenic and tumorigenic *in vitro*, and suppression of ALDH1 leads to lower tumorigenicity. Moreover, dissociated cells of engraftments created from ALDH1A1-positive cancer cells present an average of 29% ALDH1A1-negative cancer cells, indicating that the ALDH1 phenotype gives rise to heterogeneous tumors. Although ALDH1-positive breast cancer cells are believed to be directly responsible for cancer cell growth *in vitro*, the association of the ALDH1 phenotype with tumor cell proliferation *in vivo* has not been evaluated until now.

In the present study, positive ALDH1A1 expression was observed in 63.0% (92 of 146) of human invasive ductal carcinoma tissues (including slight, moderate, and strong staining), and the incidence of moderate or strong staining were 21.4%. Our observed percentage of cancer samples positive for ALDH1A1 was consistent with findings in other types of prostate, head-and-neck solid malignancies, but higher than the subpopulations of ALDH1A1-positive cells in bladder and lung tumors [26-29]. Earlier studies have reported increased ALDH1A1 expression in 30% of breast tumor specimens and 34% of inflammatory breast carcinomas [13,15], while a recent report showed the presence of epithelial ALDH1 and expanded stromal ALDH1-positive cells in 43% and 69% of breast tumor biopsies, respectively [30]. These differences among the studies may be attributed to the diversity of detection techniques and samples under investigation, especially since all the cases examined in our study were invasive ductal carcinoma. The data provides further evidence of enrichment of ALDH1-positive cancer cells in invasive ductal carcinoma tissue.

Clinical description of the ALDH1 phenotype in tumor cells is interesting. While elevated ALDH1A1 expression in tumor cells is reported to correlate with advanced tumor grade and stage in bladder and lung cancer, and patients with the ALDH1 phenotype in tumors display higher recurrence and shorter survival rates [28], tumor cell ALDH1 expression is significantly correlated specifically with triple negativity features or HER2 tumor types in the adjuvant series and tumor grade in the neoadjuvant cohort, and no significant enrichment for ALDH1 positive cells has been observed in postneoadjuvant therapy specimens, compared to pretreatment samples [30,31]. These findings suggest that the tumor microenvironment plays a role in determining the prognostic impact of stem/progenitor cells in human breast cancer [32]. Moreover, our data revealed no association of the ALDH1 phenotype with age, TNM stage, tumor size, or lymph node involvement. Surprisingly, the association of ALDH1 expression in breast cancer cells with early local recurrence affair seemed practically different with the linkage between ALDH1 phenotype and metastatic event. We observed a significant positive relationship between ALDH1 phenotype and early local recurrence affair in the patients, indicating that ALDH1-positive cases have an enlarged cancer stem cell component.

Based on our finding that cases with early local recurrence and distant metastasis show significantly more frequent epithelial ALDH1 expression, association of the ALDH1 phenotype in breast cancer with tumor cell proliferation was further evaluated in the present study. The results disclosed a positive relationship between ALDH1 phenotype and Ki67 in invasive ductal carcinoma specimens. Since the Ki67 protein is present during active phases of the cell cycle, our data suggest that the ALDH1 phenotype in tumor cells may be associated with cell proliferation. On the other hand, we observed no association between phenotypes of P53, indicative of apoptosis, and ALDH1 in invasive ductal carcinoma specimens. Thus, it appears that ALDH1 does not contribute to tumor apoptosis. Given the association between high ALDH1 expression and elevated staining for the proliferating cell marker, we focused on whether ALDH1 is linked to the cell proliferation pathway. Both immunohistochemistry and real-time PCR experiments demonstrated a strong association of ALDH1 with the reversed NOTCH1 expression in breast cancer. Although several studies showed that NOTCH family member levels were elevated in various breast tumor samples and cell lines and upregulation of specific NOTCH proteins would lead to increased tumor cell proliferation and invasion [33-35], our findings demonstrated that moderate or high expression of NOTCH1 was parallel to the absence or little expression of ALDH1 expression and this inconsistency seemed NOTCH signaling pathway may played a negative role on ALDH1-positive breast cancer cells, which was similarly with the finding that NOTCH signaling might serve as a tumor suppressor in some solid tumors [21,22]. Thus, clinical benefits by regulation of NOTCH potentially in targeting ALDH1-positive breast cancer cells may be a complex question worth researching.

Although we speculate that ALDH1 in invasive ductal carcinoma tissue contributes to tumor cells proliferation, other aspects involved in metastasis, such as drug resistance, tumor metabolism, angiogenesis, and lymphangiogenesis, have not been directly investigated to date. Notably, high ALDH1-expressing breast cancer cells survived chemotherapy/radiotherapy, relative to cells expressing low levels of ALDH1, and pre-treatment of cell populations with the ALDH inhibitor, diethylaminobenzaldehyde, resulted in significant initial sensitization of ALDH-expressing cells to chemotherapy or radiotherapy. These findings indicate that the ALDH1 phenotype contributes to both chemotherapy and radiation resistance in breast cancer [32]. More importantly, in addition to the epithelial ALDH1 phenotype, stromal ALDH1 may be associated with breast cancer development [30]. While the biological function of the ALDH1 phenotype in breast cancer has been established, the mechanisms by which ALDH1 integrates its activity to control specific events remain to be clarified. Moreover, we are yet to determine whether modulation of such a pleiotropic pathway can serve as a potential therapeutic target in breast cancer therapy and regenerative medicine.

## Conclusions

We observed variations in the prevalence of ALDH1-expressing tumor cells among different subtypes of invasive ductal carcinoma. Our findings demonstrate that the cellular subcomponent with stem cell characteristics expressing ALDH1 contributes to early tumor replase behavior, possibly in association with the NOTCH signaling pathway. In conclusion, the current study has highlighted the importance of the ALDH1 status in translating cancer stem cell research into clinical practice, and further identified ALDH1 as a potential therapeutic target in invasive ductal carcinoma.

## Abbreviations

ALDH1: Aldehyde dehydrogenase; CSC: Cancer stem cell; DAB: 3,3'-Diaminobenzidine; DAPI: 4,6-Diamidino-2-phenylindole; DFS: Disease-free survival; EDTA: Ethylene diamine tetraacetic acid; EFS: Event-free survival; ER: Estrogen receptor; FITC: Fluorescein isothiocyanate; IQR: Interquartile range; HER: Human epidermal growth factor receptor; IHC: Immunohistochemistry; LN: Lymph node; NTP: Nucleoside triphosphate; PBS: Phosphate buffered saline; PCR: Polymerase chain reaction; PR: Progesterone receptor; SP: Side population; SR: Steroid receptor; RNase: Ribonuclease.

## Competing interests

The authors declare no competing interests.

## Authors’ contributions

YL and YZ participated in the design of the study, evaluated the immunostaining and real-time PCR results, performed statistical analyses, and drafted the manuscript. CW, XZ, YX, and SS assisted with the statistical analysis and immunohistochemical and real-time PCR experiments, respectively. QS conceived the study, participated in its design, and helped to draft the manuscript. All authors read and approved the final manuscript.
